# Accuracy of Automatically Identifying the American Conference of Governmental Industrial Hygienists Threshold Limit Values Twelve Lifting Zones over Three Simplified Zones Using Computer Algorithm

**DOI:** 10.3390/s25010111

**Published:** 2024-12-27

**Authors:** Menekse S. Barim, Ming-Lun Lu, Shuo Feng, Marie A. Hayden, Dwight Werren

**Affiliations:** 1 National Institute for Occupational Safety and Health, Cincinnati, OH 45226, USAnqx6@cdc.gov (M.A.H.);; 2 Meta, San Jose, CA 94025, USA; f3e7n2g1@gmail.com

**Keywords:** ACGIH TLVS for lifting, IMU, lifting, computer algorithm

## Abstract

The American Conference of Governmental Industrial Hygienists (ACGIH) Threshold Limit Values (TLVs) for lifting provides risk zones for assessing two-handed lifting tasks. This paper describes two computational models for identifying the lifting risk zones using gyroscope information from five inertial measurement units (IMUs) attached to the lifter. Two models were developed: (1) the ratio model using body segment length ratios of the forearm, upper arm, trunk, thigh, and calf segments, and (2) the ratio + length model using actual measurements of the body segments in the ratio model. The models were evaluated using data from 360 lifting trials performed by 10 subjects (5 males and 5 females) with an average age of 51.50 (±9.83) years. The accuracy of the two models was compared against data collected by a laboratory-based motion capture system as a function of 12 ACGIH lifting risk zones and 3 grouped risk zones (low, medium, and high). Results showed that only the ratio + length model provides acceptable estimates of lifting risk with an average of 69% accuracy level for predicting one of the 3 grouped zones and a higher rate of 92% for predicting the high lifting zone.

## 1. Introduction

In the workforce, musculoskeletal pain is very common and costly, with incidents related to work activities [1]. One of the most common types of musculoskeletal pain is low back pain (LBP). Among health problems causing disability in the United States, LBP is ranked second, with more than 80% of people experiencing LBP at one point in their lives [2,3,4]. Work involving heavy and repetitive manual lifting maBy increase the risk of low back disorders (LBDs) [5]. To prevent LBDs, accurate quantifications of risk factors are imperative [6,7].

**Figure 1:**
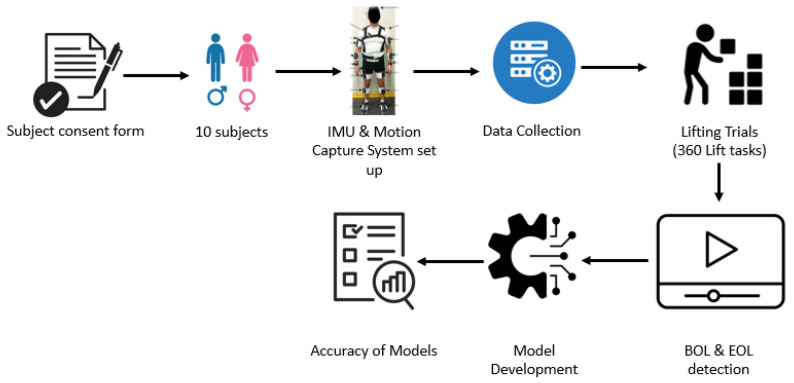
Methodology flowchart.

The American Conference of Governmental Industry Hygienists (ACGIH) developed Threshold Limit Values (TLV) for lifting based on the Revised NIOSH Lifting Equation (RNLE), which categorizes twelve zones. These zones were developed relative to the participants’/subjects’ bodies at which various maximum lifting loads are defined (Figure 1). These zones can be simplified into groups of low, medium, and high-risk zones to provide more general guidelines for safe lifting that can be applied without careful measurement of lifting factors [8,9].

ACGIH TLV for lifting is a lifting risk assessment method that is based on biomechanical, psychophysical, and epidemiological information [10]. The ACGIH TLV for lifting provides practical guidelines for minimizing the risk of lifting associated with LBDs and shoulder problems [11].

The ACGIH TLV method considers lifting risk factors such as the vertical height of the lift, horizontal distance of the lift, lift frequency and duration (LD), and the load of the weight [12]. The horizontal distance (H) is defined as the projected distance on the transverse plane from the center of two ankles to the center of two hands. The vertical height (V) is defined as the height from the center of two hands to the ground. The H and V variables are critical factors for using the ACGIH TLV for lifting to determine the maximum allowable weight for a given lift. However, taking measurements for the two variables in the field may interrupt the worker’s job performance and may be tedious for variable lifting tasks [13].

Using wearable inertial measurement unit (IMU) sensors in the field for automatically determining the hand location of lifting tasks may provide a practical solution to reducing the work interruption for using the ACGIH TLV for lifting [8,14,15,16]. IMUs have been used to assess postural risk for musculoskeletal disorders in several occupations [17,18]. The ability of the IMU sensors to detect unsafe lifting tasks in real-time would provide significant benefits to employers and safety managers for risk control. The accuracy levels of IMU-based systems for ergonomic assessments to estimate body postures have been previously studied [13,19,20,21]. Generally, the number of sensors is an important indicator of the accuracy of measuring whole-body posture. The number of sensors significantly influences the accuracy of full-body posture measurements. Detailed biomechanical models typically use 13 to 17 sensors mounted across body segments, such as the Xsens MVN tracking system, which requires 17 sensors [22]. However, high costs, time-consuming setup, and required software expertise pose challenges for field use. A prior study used five sensors to record movements in six subjects, including walking, jumping, and lunging [23]. Their findings suggest that a five-sensor setup may be sufficient to differentiate gross movement activities and reconstruct body posture effectively. However, it is unknown whether the five IMU sensor systems are capable of measuring detailed lifting risk factors for LBDs, such as the V and H for the ACGIH TLV for lifting.

We previously developed machine learning and equation-based hybrid approaches to estimate V and H [8,13,16]. Expanding on these previous studies, we investigated the accuracy of predicting the ACGIH 12 lifting zones and 3 grouped lifting zones to represent an industry common practice of using red (high), yellow (medium), and green (low) as three levels of risk for LBDs.

## 2. Materials and Methods

An overall methodology flowchart can be found in Figure 1.

### 2.1. Experimental Setup

**Figure 2:**
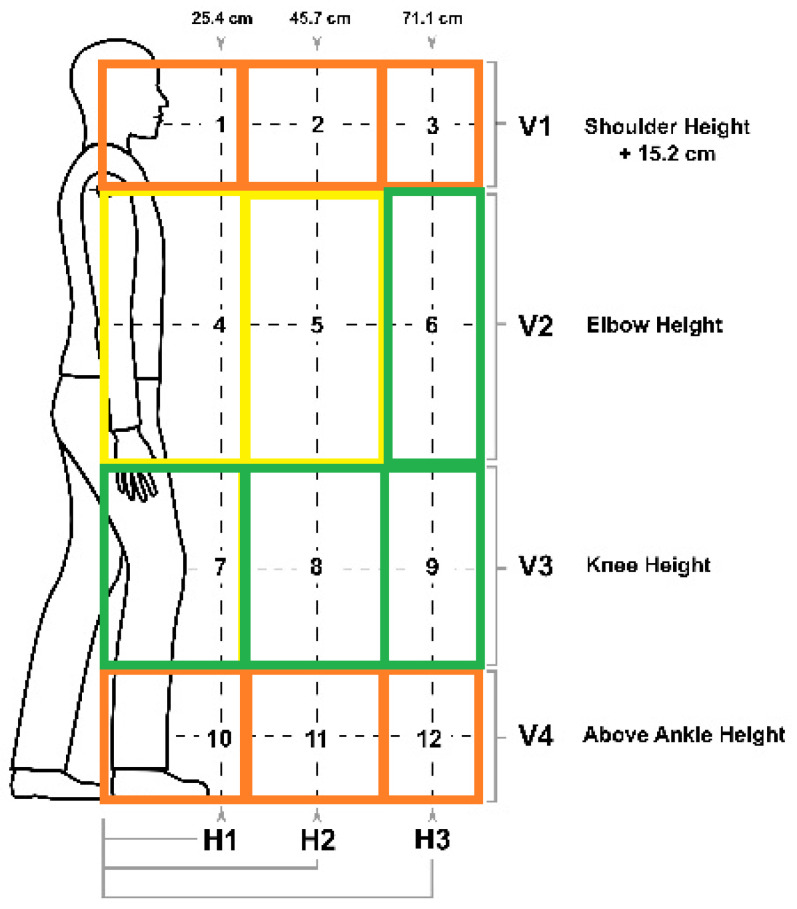
Initial lifting positions based on the ACGIH TLV for lifting (H1: near horizontal distance from the object being lifted (wired grid), H2: middle distance, H3: far distance, V1: shoulder height, V2: elbow height, V3: knee height, and V4: above ankle height) (yellow indicates low-risk zones (4 and 5), green represents medium-risk zones (5, 7, 8, and 9), while orange signifies high-risk zones (1, 2, 3, 10, 11, and 12)).

**Figure 3:**
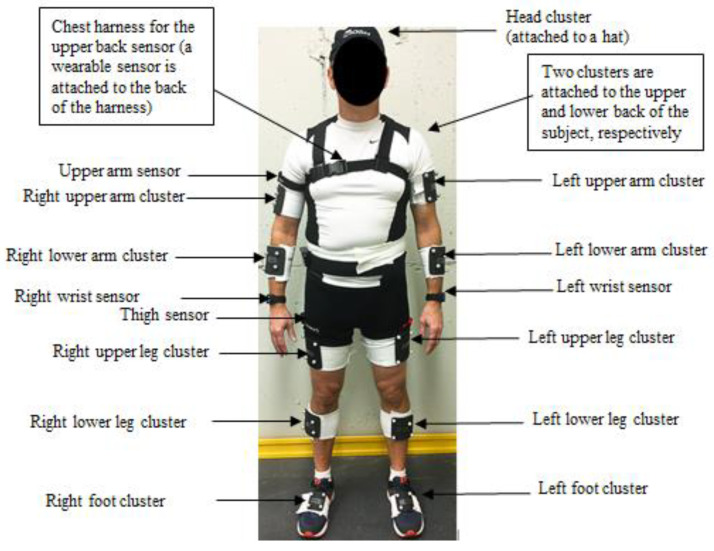
Placement of the IMU sensors and marker clusters.

Data were collected on ten subjects (5 females and 5 males) at the Taft Laboratories, National Institute for Occupational Safety and Health (NIOSH), in Cincinnati, Ohio. The average (standard deviation) age of participants was 51.50 (9.83) years. Colleagues who had musculoskeletal disorders or pain (now or in the past three months), were under age 18, were pregnant, or were in the principal investigator’s work unit were excluded. This study was reviewed and approved by the NIOSH IRB ^§§^ (^§§^ See 45 C.F.R. part 46; 21 C.F.R. part 56—protocol # 16-DART-05XP). Subjects performed symmetrical lifting tasks in 12 risk zones (Figure 2) defined by the ACGIH TLV for lifting. Subjects were asked to start 12 symmetrical lifting tasks, one in each of these risk zones. These tasks were repeated three times, resulting in 36 lifting trials for each subject. These trials were randomly assigned to each subject to avoid training effects. Prior to the first lifting trial, subjects wore five IMU sensors (Kinetic Inc., New York, NY, USA) and one data logger clipped on the right side of the waist (Figure 3). Thirteen IR reflective marker clusters were attached to their body segments for a laboratory-based optical motion capture system (OptiTrack Inc., Corvallis, OR, USA, and Innovative Sports Training, Inc., Chicago, IL, USA). Each IMU sensor consisted of a tri-axial gyroscope and accelerometer sampling at a rate of 25 Hz. The motion capture and IMU sensor data were synchronized prior to the first trial.

A wire grid [13] was used to simulate tote box lifting during the trials. The grid was placed on a platform to establish the initial lifting height. A height-adjustable pole was utilized to target various lifting positions throughout the trials. These positions were adjusted based on the subjects’ height following the ACGIH TLV Lifting Zones. Three designed H zones (25.4, 45.7, and 71 cm from the center of the ankles) guided subjects’ lifts, with adjustments made for realistic motions in certain zones. Pre-determined foot and hand placements ensured proper lifting postures across 12 zones, and subjects carried the box from the adjusted starting positions, which were tailored to their anthropometric measurements, to the endpoint.

### 2.2. Development of Models

To process the IMU data, we implemented a hybrid model combining machine learning and trigonometric analysis to accurately detect lifting motions and assess hand positions in relation to the body. Two key modules were developed for this purpose. The first, the lifting detection module, identified the start and endpoints of lifting events. Simultaneously, the sensor fusion module tracked device orientations in three dimensions, correcting gyroscope drift to improve accuracy. To simplify sensor preparation, a ratio model was introduced, using forearm length to estimate other body segment lengths, ensuring the system could compute essential variables with minimal setup. This innovative approach not only streamlined sensor placement but also enhanced real-time orientation estimation during dynamic work environments.

**Figure 4:**
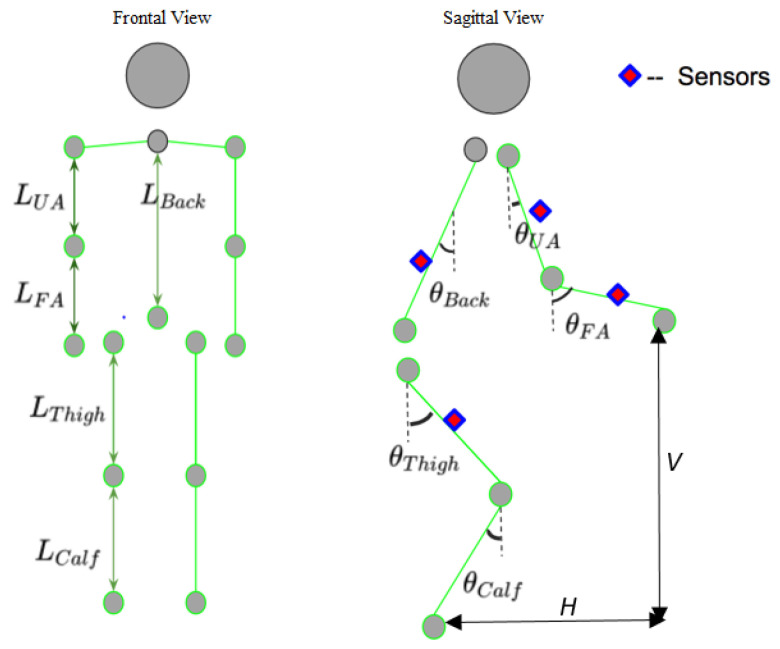
Body length ratio model and angular data of four sensors used for estimating V and H.

Figure 4 shows the ratios of the body segments, and the angle θ used to calculate the V and H variables was derived from the kinetic accelerometer data, as processed and filtered through sensor fusion, rather than the gyroscope data:

$$V=L_{back}\times \cos\left(\theta_{Back}\right)+L_{Thigh}\times \cos(\theta_{Thigh}){+L_{calf}-L}_{UA}\times \cos(\theta_{UA})-L_{FA}\times \cos(\theta_{FA})$$

$$H=L_{UA}\times \sin(\theta_{UA})+L_{FA}\times \sin(\theta_{FA})+L_{Back}\times \sin(\theta_{Back})-L_{thigh}\times \sin(\theta_{Thigh})$$

To enhance the accuracy of the ratio model, we developed a second model, referred to as the ratio and length model, which incorporated individual anthropometric measurements, such as the forearm, upper arm, back, thigh, and calf lengths. These measurements, combined with the same gyroscope data used in the ratio model, improved precision in calculating lifting risk variables (H and V). The length of the calf (Lcalf) was considered to have minimal influence on the calculations. Pre-determined lifting zones, adjusted using ankle height for lower levels, were incorporated based on subject-specific measurements. We assumed Lcalf has little effect because we did not use calf measurement during the lifting trial. Therefore, this angle was ignored in the above equations.

### 2.3. Accuracy of Models

The accuracy of both models was evaluated by comparing the estimated hand positions in the 12 lifting zones with data gathered from a laboratory motion capture system. Since the initial lift position was customized based on the subject’s anthropometric data, and foot placement in horizontal zones varied slightly between trials, true lifting zones fluctuated. Motion capture data were used to define these zones for accuracy assessment. The analysis of V and H data collected by the motion capture system showed that V was categorized into four vertical height ranges (V1: 1.45–1.70 m, V2: 1–1.45 m, V3: 0.5–1 m, V4: 0–0.5 m) and H into three horizontal distances (H1: 0–0.25 m, H2: 0.25–0.457 m, H3: 0.457–0.712 m). These ranges were then applied to assess the accuracy in estimating (1) horizontal hand positions (H) within the three zones, (2) vertical hand positions (V) in the four zones, (3) all 12 zones, and (4) the three grouped lifting zones based on recommendations from the Los Alamos National Laboratory [8].

The accuracy of zone identification by the IMU system was measured by comparing it with the laboratory motion capture system. For example, if the estimated hand location by the wearable system was zone 12 rather than zone 11, estimated by laboratory motion capture data, the accuracy was zero. When the zone identified by the IMU system aligned with the one determined by the laboratory motion capture system, the accuracy was 100%. The final accuracy calculation was an average of all the trials’ accuracy measurements. Essentially, the final accuracy reflected the percentage of correctly identified zones across all trials.

## 3. Results

### 3.1. Accuracy of 12 Lifting Zones

**Figure 5:**
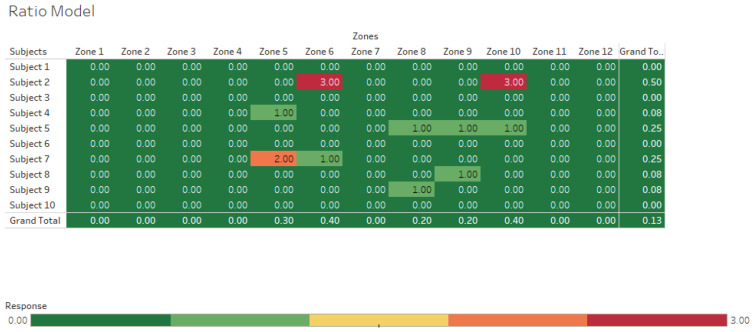
Ratio model heatmap showing correlations between lifting zones identified by computer models using data from inertial measurement units vs. a laboratory-based motion capture system. A value of 0 indicates no correlation, while a value of 3 signifies 100% correlation for that specific zone over all 3 trials for a given subject.

**Figure 6:**
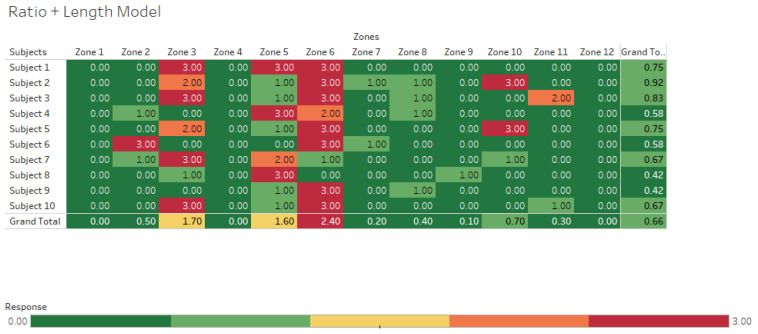
Ratio + length model heatmap showing correlations between lifting zones identified by computer models using data from inertial measurement units vs. a laboratory-based motion capture system. A value of 0 indicates no correlation, while a value of 3 signifies 100% correlation for that specific zone over all 3 trials for a given subject.

To visualize the accuracy of hand locations estimated by the two models, clustering heat maps of the estimated hand locations by the IMU system are presented in Figure 5 and Figure 6 for the ratio and ratio + length models, respectively. The cluster heatmap consists of a rectangular tiling in which each tile is shaded on a color scale to represent the value of the corresponding element of the data matrix. The goal of using a heatmap was to provide a comprehensive view of the data. Correlation values scaled between 0 (no correlation) and 1 (perfect correlation) could be seen in heat maps. The heatmaps illustrate the correlation values derived from each subject’s trial across different zones. Each subject completed three trials for each zone, allowing us to examine the correlation between those trials. In this context, a correlation value of 0 indicates no correlation between the trials, while a value of 3 signifies a 100% correlation for that specific zone over all three trials for a given subject. This means that for that subject, the model accurately detected the zone based on their trial data. In these figures, darker red colors indicated higher accuracy, whereas green colors represented less accuracy; for example, in Figure 4, it was clear that the accuracy of estimating each zone was pretty low, which is 4% (15/360) in the ratio model. However, this accuracy was increased to 22% (78/360) in the ratio + length model. The heatmaps highlight accurately determined zones with correlation values of 0, 1, 2, and 3. When these values are summed for the ratio model, only 15 trials were accurately identified out of 360 (Total trials = 10 subjects × 3 trials/subject × 12 zones = 360 trials). However, the accuracy improved significantly in the ratio + length model, where the number of accurately determined trials increased to 78 out of 360, resulting in an accuracy of 22%.

**Figure 7:**
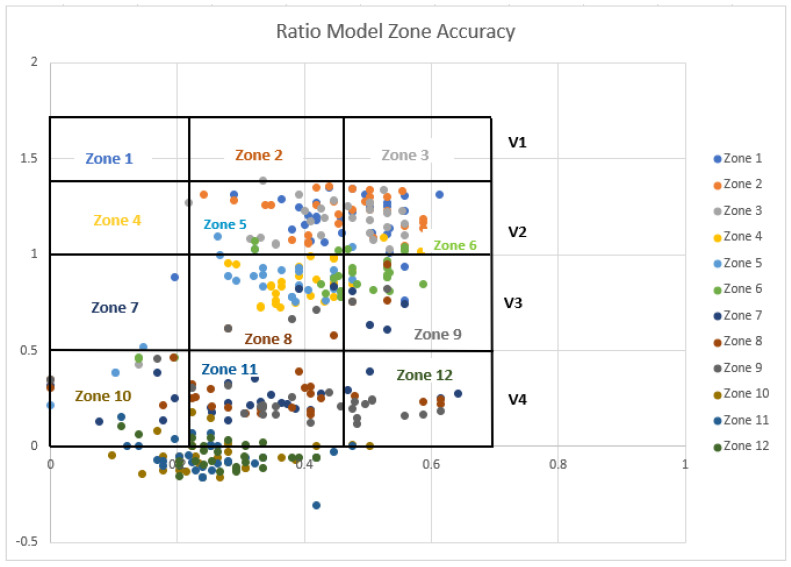
Scatter plot of the ratio model dots represents lifting zones identified by the computer model using data from inertial measurement units, and the grid represents lifting zones identified through a laboratory-based motion capture system. Matching colors between dots and zone labels represents the correlation.

**Figure 8:**
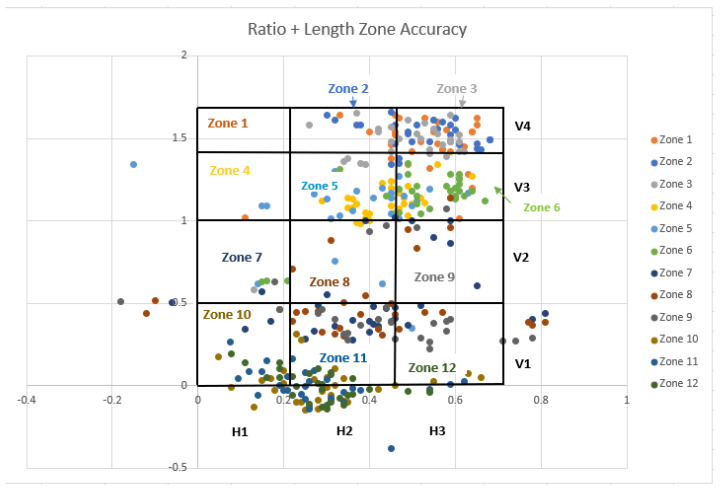
Scatter plot of the ratio + length model dots represents lifting zones identified by the computer model using data from inertial measurement units, and the grid represents lifting zones identified through a laboratory-based motion capture system. Matching colors between dots and zone labels represents the correlation.

In addition to the heat maps, scatter plots were also created for both the ratio and ratio + length models. The main purpose of creating scatter plots was to differentiate each zone separately in the graph and visualize how well each algorithm detected the actual zone. The vertical height (V) is represented by the *Y*-axis, and the horizontal distance (H) is represented by the *X*-axis. The twelve established lifting zones were delineated by the V_1–4_ and H_1–3_ lines. In Figure 7 and Figure 8, the accuracy of each zone is depicted with different colors, aligned with predefined vertical and horizontal values. The color-coded zones correspond to the text color for each zone, allowing for easy identification of accuracy levels. For instance, zones 1, 2, and 3 all displayed 0% accuracy, indicating that none were correctly identified by the algorithm. Specifically, the points for zone 1 were misclassified as zone 5, while zone 2 was identified as both zone 5 and zone 6, and zone 3 was also recognized as zone 5 and zone 6. By following the matching colors of the dots and the zone text, you can determine the accuracy of the classifications. When zone 1 was analyzed in the ratio + length model, the accuracy was also 0%. However, the accuracy levels were increased in other zones in the ratio + length model.

Results of the accuracy analysis showed that the ratio model (Figure 6) had a 37% accuracy in estimating H, a 14% accuracy in estimating V, and a 4% accuracy in estimating each zone. The mean error of V measurements between the motion capture system and the ratio model was −33 cm. The mean errors of V for the four vertical zones V_1_–V_4_ were −43, −30, −28, and −31 cm, respectively. The lowest error was observed in V_3_, which was the height between knuckle and mid-shin heights. The mean error of H measurements between the motion capture system and the ratio model was −6.2 cm. The mean errors of H for the three horizontal zones H_1_–H_3_ were −12, −7, and −23.5 cm, respectively. The performance of the ratio model for measuring H was poor. This poor performance significantly affected the overall accuracy.

This poor performance may be attributed to increased variability in horizontal (H) estimations due to the complexity of capturing and modeling distances across zones with differing characteristics. For example, the larger error in H_3_ could result from this zone involving greater distances from the motion capture reference points, which likely amplified measurement inaccuracies. Additionally, participant movement or positioning during data collection may have introduced further variability, particularly in zones requiring extended reach.

As shown in Figure 7, the accuracy of the ratio + length model improved for certain measures. Vertical height estimates saw significant improvement, with 61% accuracy compared to 14% for the ratio model. However, the accuracy of estimating horizontal distances dropped slightly to 34%. Overall, 28% of the lifting zones were accurately identified.

The mean error between the motion capture system and the ratio model was −14 cm. The mean errors of V for the four vertical zones V_1_–V_4_ were −14, −4, −12, and −28 cm, respectively. The lowest error was observed in V_2_, which was the height between the shoulder and waist. The mean error of H measurements between the motion capture system and the ratio model was −2.2 cm. The mean errors of H for the three horizontal zones H_1_–H_3_ were −18, −0.9, and −19 cm, respectively. By incorporating subjects’ anthropometric measurements, the performance of the ratio + length model was significantly improved compared to the ratio model. However, the overall performance was still affected by the horizontal location of the lift from the middle zones.

### 3.2. Accuracy of Three Grouped Lifting Zones

Los Alamos National Laboratory simplified the ACGIH zone by classifying it into low-risk, medium-risk, and high-risk zones. In their version, only zone 4 was considered low-risk, zones 1, 5, 7, 8, and 10 were identified as medium-risk, and zones 2, 3, 6, 9, 11, and 12 were classified as high-risk. We modified this classification slightly in our approach. In our model (shown in Table 1), zones 4 and 5 are categorized as low-risk, zones 6, 7, 8, and 9 as medium-risk, and zones 1, 2, 3, 10, 11, and 12 as high-risk. This adjustment allows for a better fit for our analysis and risk assessments.

The three standard lifting zones were commonly used by safety and health professionals to guide employees on how to safely position loads during lifting.

The accuracy of hand locations estimated by the two models was compared in Table 2 and Table 3 for the ratio and ratio + length models, respectively. Twelve lifting zones were simplified into three zone categories: low-risk zones, medium-risk zones, and high-risk zones, as identified in Table 1.

In this process, normalization was applied to categorize the outputs from two different systems—a motion capture system (MM) and a computer algorithm (FM)—into three risk zone categories: low, medium, and high. Normalization was applied to account for the different ranges of the risk zones:

- For low-risk zones: Any zone that was classified as low–low, low–medium, or low–high by MM–FM was grouped into the overall low category. The total number of estimated zones in this combined low-risk category was 60.
- For medium-risk zones: Similarly, any zone classified as medium–medium, medium–low, or medium–high was counted in the medium category. The total number of estimated zones in this medium-risk category was 120.
- For high-risk zones: Zones classified as high–high, high–medium, or high–low were combined under the high category, with a total of 180 estimated zones.

Since the high-risk zone category was three times larger than the low-risk zone, the disparity could have influenced the results. To mitigate this, each zone was normalized to bring all variables into the same range, ensuring comparability across risk categories. In order to evaluate the proposed method’s performance compared to other techniques, several metrics were calculated. Two primary metrics were used for an understanding and measure of accuracy: precision (positive predictive value) and recall (sensitivity). The precision-recall metrics framework is widely used and recognized as a benchmark for evaluating performance in information retrieval [24]. Precision, recall, and F-score are commonly employed to assess image segmentation, particularly for boundary detection [24]. Precision measures the proportion of positive classifications that are correct, while recall reflects the likelihood of correctly identifying true boundary pixels. For this analysis, we treated values from the FM system as subject ratings and values from the MM system as the gold standard. The F-score represents the harmonic mean of precision and recall, providing a balanced evaluation [25].

Table 2 provides a performance analysis of the ratio model and laboratory motion capture data with precision, recall, and F-score values. You can see that precision was simply the ratio of correct positive predictions out of all predictions made. The overall recall is 0.30, representing the average accuracy across the three zones: low–low (0.07), medium–medium (0.33), and high–high (0.51). For instance, when we look at the low zone accuracy, there were 7 correct positive predictions for low-risk zones out of 31 positive predictions with a precision level of 0.22. The recall value for predicting a low-risk zone was 0.07. The F-score (weighted average of precision and recall) was 0.1. Like precision and recall, a poor F-score was 0.0, and a perfect F-score was 1.0. Each row total is 1 for the laboratory motion capture data because each lifting risk zone (low, medium, and high) was divided equally. However, for the ratio model, each row was not equal to 1 because the number of estimated real zones was not equal for each zone.

In both Table 2 and Table 3, you can find the values for positive predictions. Cells highlighted in blue represent higher prediction values.

The same analysis of the model performance measures was performed for the ratio + length model (Table 3). The overall recall is 0.63, representing the average accuracy across the three zones: low–low (0.07), medium–medium (0.33), and high–high (0.51).

The ranges were as follows: The low zone ranged from 0 to 51, the medium zone ranged from 0 to 128, and the high zone ranged from 0 to 181. They all normalized to the same range, and the results showed that the ratio + length model had a 63% overall recall. In this analysis, there were 51 correct positive predictions for low-risk zones out of 59 positive predictions, with 0.86 for precision, which was very high, which means 86% of the predictions were conducted correctly. When we looked at the recall value for predicting a low-risk zone, it was 0.51. This value was good because it was more than 0.5. For the F-score, which is the weighted average of precision and recall, it was 0.64, which was good. Like precision and recall, a poor F-score was 0.0, and a perfect F-score was 1.0.

Upon closer examination, 7% of the lifting trials showed uneven motion, and 8% of the motion data were missing. Since the missing data were estimated using nearby proxy information, this likely increased errors in determining the correct lifting zones.

## 4. Discussion

This paper describes the accuracy of two computational models using five IMU sensor data for estimating the lifting zones of the ACGIH TLVs for lifting. In these two models, the determination of lifting zones relies on V and H measurements. A previous study [13] showed that the same computational models for measuring the actual distances of V and H produced large errors. Poor performance was observed in the ratio model, with mean errors of 33 cm and 6.5 cm for V and H, respectively. However, the mean errors were reduced to 14 cm and 2.2 cm for V and H in the ratio + length model, respectively. The body segment length ratios used in the ratio model were based on the population means of the body segments to simplify the data collection process for the wearable system [26]. Clearly, the inclusion of the precise measurements of the body segment lengths improved the model’s accuracy from 4% to 34% for individual zones, 14% to 61% for V zones, and 30% to 63% for three grouped risk zones. However, the accuracy level of H did not improve, with 37% for the ratio model and 34% for the ratio + length model.

Our study aligns with findings from other IMU-based research, particularly in how we mitigate the effects of magnetic disturbances and gyroscopic drift by focusing on inclinometry rather than spatial orientation. For example, ref. [27] noted that “IMUs provide reliable data in controlled environments, but magnetic disturbances can significantly affect the accuracy of spatial orientation measurements.” In their work, similar to ours, they emphasized the challenge of maintaining accuracy due to gyroscopic drift, especially over extended periods. Similarly, ref. [28] observed that “while segment-based inclinometry reduces the impact of magnetic interference, it does not completely eliminate positional drift,” particularly during prolonged use in clinical gait analysis. This mirrors our findings where inclinometry, while effective, still resulted in some positional errors. By comparing our results with these studies, it becomes evident that while inclinometry helps alleviate some limitations inherent to IMUs, particularly related to magnetic disturbance and gyroscopic drift, these devices still face challenges in environments prone to interference or in tasks requiring high precision over time. Acknowledging these limitations allows for a more nuanced understanding of the capabilities and constraints of IMU technology in ergonomic assessment and motion capture.

The findings of this study (see Figure 7 and Figure 8) show that both ratio and ratio + length models had zero accuracy for estimating the zone V_1_. The inability of the models to estimate the location of the hands in V_1_ primarily resulted from inaccurate projection of the gyroscope data from the arm sensors on the sagittal plane. The V_1_ zone was the area where subjects needed to perform an overhead lift. In this position, subjects were observed to pronate their upper or lower arms in order to initiate the lifting task.

This pronation altered the orientation of the gyroscopes relative to gravity, introducing variability that the two-dimensional computation models could not adequately account for. Specifically, the models assumed consistent alignment of gyroscope data with the sagittal plane, which was disrupted by the rotational movement of the arms. As a result, the projection of hand location was skewed, leading to a complete failure in accurately estimating V1.

To address this limitation, future work could explore integrating additional sensor modalities, such as magnetometers or optical trackers, to improve orientation accuracy during arm pronation. Alternatively, refining the computational algorithms to dynamically adjust for variations in arm positioning relative to gravity could help mitigate these errors. Such enhancements would be critical for improving the model’s performance in overhead lifting tasks and ensuring greater accuracy across all vertical zones.

In addition, as shown in Figure 7 (ratio model), when the actual zone was 1, 2, or 3 (above shoulder height), the algorithm incorrectly detected the lifting risk zones as 4, 5, and 6 (mostly shoulder and mid-shin heights). This was similar to the ratio + length model, as shown in Figure 8, as the system did not detect anything in zone 1. The inability to detect zone 1 resulted from inaccurately estimated hand locations in H1 and H2 for lifting trials in the V1 zone. Similarly, inaccurately estimated hand locations in H2 and H3 for lifting trials in the V4 zone caused data to cluster in zones 10 or 11. The inaccurate estimations of the hand location were most likely caused by subjects’ uneven lifting motion. The assessment of the hand locations was further compromised by a small portion of missing laboratory motion data due to missing markers obstructed by the subjects’ bodies during lifting trials. These misclassifications affected the precision of zone identification, especially in zones where lifting height was critical (e.g., V4), and emphasized the need for more precise hand location detection. Future studies could mitigate this by incorporating additional sensors or improving sensor placement to reduce the likelihood of missing data and improve hand location accuracy.

The accuracy calculation for the 12 zones did not account for estimates that fell near the boundaries of the correct V, H, and individual zones, resulting in a zero-accuracy score in these cases. However, the actual difference between the correctly and incorrectly estimated zones could be small, just a few centimeters, as shown in Figure 7 and Figure 8. This classification error may be less critical if the actual estimated distances for V and H are used as the outcome measures instead.

Most practical risk assessments in the field employ three simplified lift zone categories (low (yellow), medium (green), and high (red)) to suggest corrected actions [8]. The 12 ACGIH TLV zones were grouped into 3 lifting risk zone categories in this study for this practical reason. In order to compare the accuracy of the 3-zone system, 3 × 3 matrices were explored, and each range for each zone was normalized. Results showed that the simplified three-lifting-risk-zones approach improved the overall accuracy of the wearable sensor system. The accuracy level of the 12-zone system for the ratio model was 4%, and for the ratio + length model, it was 28%. The accuracy level of the 3-zone system was 30% for the ratio model and 63% for the ratio + length model. The improved accuracy levels of the 3-zone system most likely resulted from the adjustments for the estimates of the hand locations that fell near the boundaries of the correct V, H, and individual zones.

To understand the risk classification information further, each of the three risk zones (low, medium, and high) was classified as low–low, low–medium, and low–high, where the first and second words represent the classification information by the wearable (IMU-based computer algorithm (FM)) and gold standard (motion capture (MM)) systems, respectively. As can be seen in Table 2, the accuracy levels of the ratio model varied differently across the three risk levels. According to this calculation, the model’s accuracy rate for the low-risk zone (low–low) is 7%, the medium zone (medium–medium) is 33%, and the high zone (high–high) is 51%. For instance, when the actual zone was low, the ratio model detected it as a medium zone 88% of the time. The ratio + length model performed much better than the ratio model. This model’s accuracy rates for the low, medium, and high-risk zones were 51%, 45%, and 92%, respectively. In other words, the ratio + length model is better suited for classifying high-risk lifting zones with an accuracy of 92%.

The limitations of these models can be attributed to several factors. First, the 2D computational model was constrained by its inability to account for three-dimensional movement dynamics. Additionally, the assumption that the hands and feet remained parallel during lifting trials was often violated. Observed variations, such as forearm pronation and bent lower legs, which were not captured due to the lack of corresponding sensor data, further contributed to inaccuracies. This was particularly evident in the estimation of the V1 zone, where subjects performed overhead lifts. The arm pronation altered the gyroscope’s orientation relative to gravity, introducing variability that the models could not adequately compensate for.

Another limitation of this study is the small sample size (n = 10), which restricted the ability to generalize the findings. While a larger sample size would have provided more robust results and allowed for generalization, this study still provides valuable insights into the accuracy of the models for estimating lifting risk zones. Future research should aim to include a more diverse sample to enhance the external validity of the findings. Regarding habitual physical activity, we recognize that participants’ baseline activity levels could influence lifting performance, and thus, this could be an important factor to consider in future studies. However, the focus of this study was on evaluating the accuracy of the models based on specific lifting trials, and we did not specifically assess habitual physical activity.

These findings are consistent with other IMU-based studies [19,29,30,31,32]. IMUs measure kinematic values through relative acceleration rather than absolute coordinates, making them susceptible to drift effects that distort data. Mechanical inaccuracies, such as misalignment of the IMU modules with body contours, can exacerbate these errors. Previous studies have emphasized that recalibration algorithms could mitigate these limitations, significantly enhancing IMU measurement accuracy [33]. For instance, ref. [34] demonstrated that recalibration algorithms could supplement system shortcomings, improving reliability and precision.

Despite these challenges, the study findings suggest that the ratio + length model, using a simple system of five IMU sensors, could still be useful in identifying high-risk lifting zones with an accuracy of up to 92%. This highlights the potential of wearable IMU systems for ergonomic risk assessment. Furthermore, advancements in computational techniques could offer solutions to the current limitations. Recent research has shown that machine learning (ML) approaches can effectively classify lifting risk categories [35]. For example, ML algorithms trained on time-domain features extracted from IMU data achieved moderate to high classification accuracy. Ref. [36] reported a 65% accuracy for classifying low- and high-risk lifting activities using Linear Discriminant Analysis, while [37] achieved up to 75% accuracy for posture classification using Support Vector Machines.

Additionally, it is important to note that the accuracy of the two models for identifying the lifting risk zones using gyroscope information from five IMUs was evaluated exclusively in laboratory conditions in this study. While this controlled environment allowed for systematic evaluation of the models’ performance, real-time application would introduce additional challenges. For instance, in a real-world setting, factors such as sensor misalignment, environmental influences, and the variability of subject movement could significantly impact the accuracy and reliability of the models. These challenges were not present in the controlled laboratory environment, and therefore, the findings may not directly translate to real-time or field applications.

Future research should explore developing three-dimensional computational models or integrating ML techniques for lifting risk classification. Expanding the sensor network or incorporating additional modalities, such as magnetometers, could improve the models’ ability to capture complex movement dynamics. Refining recalibration processes and addressing limitations in sensor placement are critical for enhancing the reliability of IMU-based systems. These advancements will be crucial for improving the precision and applicability of wearable systems in real-world ergonomic assessments and worker safety evaluations.

## 5. Conclusions

The proposed ratio + length computational models using only the gyroscope data relative to the gravity from five wearable IMU sensors may provide a simple method for determining three lifting risk levels (low, medium, and high) defined by the ACGIH TLV for lifting for two-handed symmetrical lifting tasks. The application of the model for estimating 12 individual ACGIH lifting risk zones is limited due to a low average accuracy rate.

## Figures and Tables

**Table 1:** Simplified ACGIH Lifting TLV Zones.

*Low-risk Zones*	*4 and 5*
*Medium-risk Zones*	*6, 7, 8, and 9*
*High-risk Zones*	*1, 2, 3, 10, 11 and 12*

**Table 2:** Zone Category Accuracy of the Ratio Model.

Ratio + Length Model (FM)						
Laboratory Motion Capture Data (MM)		**Low**	**Medium**	**High**	**Sums**	**Avg**
	**Low**	0.07	0.88	0.05	1.00	-
	**Medium**	0.03	0.33	0.64	1.00	-
	**High**	0.22	0.28	0.51	1.00	-
	**Precision p**	**0.22**	**0.22**	**0.42**	**-**	**0.29**
	**Recall r**	**0.07**	**0.33**	**0.51**	**-**	**0.30**
	**F-score**	**0.11**	**0.26**	**0.46**	**-**	**0.28**

**Table 3:** Zone Category Accuracy of the Ratio + Length Model.

Ratio + Length Model (FM)						
Laboratory Motion Capture Data (MM)		**Low**	**Medium**	**High**	**Sums**	**Avg**
	**Low**	0.51	0.49	0.00	1.00	-
	**Medium**	0.05	0.45	0.50	1.00	-
	**High**	0.03	0.05	0.92	1.00	-
	**Precision p**	**0.86**	**0.45**	**0.65**	**-**	**0.65**
	**Recall r**	**0.51**	**0.45**	**0.92**	**-**	**0.63**
	**F-score**	**0.64**	**0.45**	**0.76**	**-**	**0.62**

## Data Availability

The datasets presented in this article are not currently available for public access because the research is still in progress. Data requests to access the datasets should be directed to oih9@cdc.gov.
